# Supplementary figures and images for: Osmotrophic glucose and leucine assimilation and its impact on EPA and DHA content in algae
Source: PeerJ. 2020 Jan 3;8:e8363. doi: 10.7717/peerj.8363 (PMC6944096; doi:10.7717/peerj.8363)

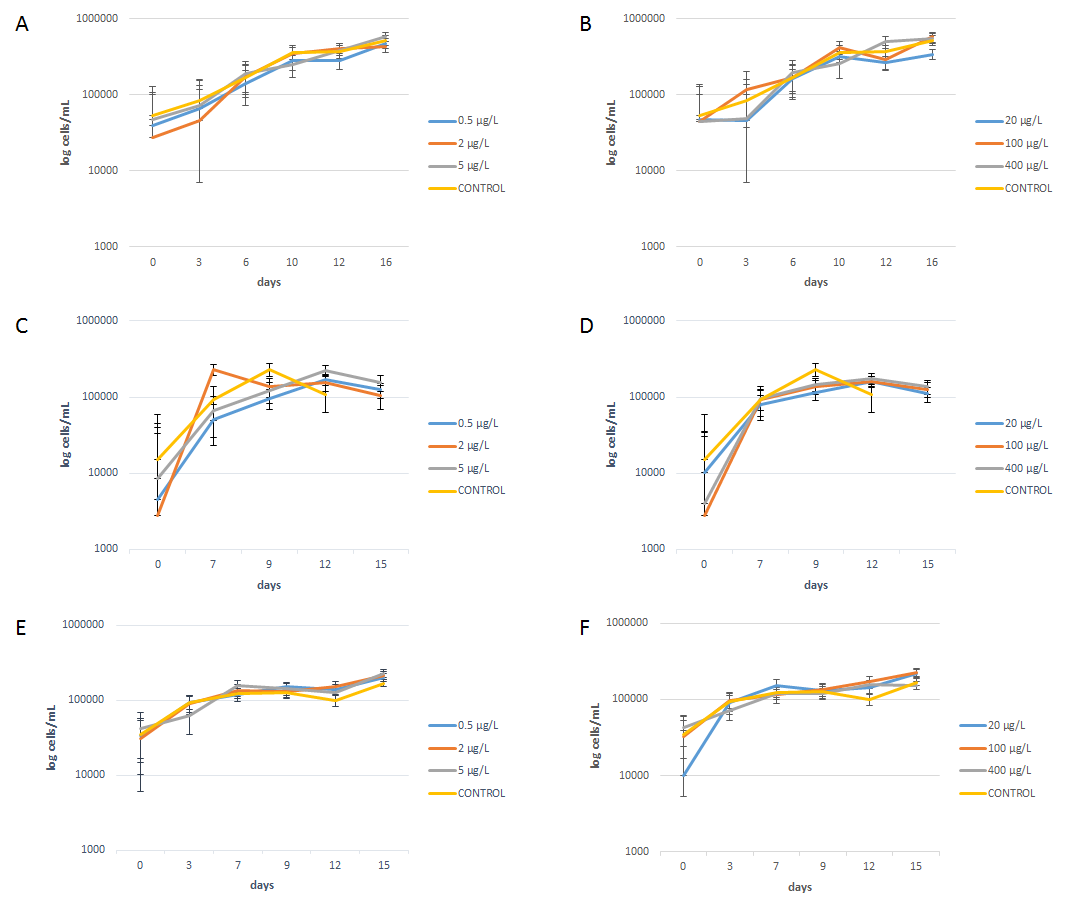

Supplement: Supplemental Information 3 — Cryptomonas sp. when grown with different concentrations of glucose (A) and leucine (B), Cryptomonas ozolinii with glucose (C) and leucine (D), and Mallomonas kalinae with glucose (E) and leucine (F). Error bars show the standard errors. [file peerj-08-8363-s003.png]
